# The Gendered Effects of Education and Acculturation on Older Korean Immigrants’ Cognitive Function

**DOI:** 10.1093/geroni/igaa057.1066

**Published:** 2020-12-16

**Authors:** Eun Young Choi, Yuri Jang, David Chiriboga

**Affiliations:** 1 Leonard Davis School of Gerontology, Los Angeles, California, United States; 2 University of Southern California, Los Angeles, California, United States; 3 University of South Florida, Tampa, Florida, United States

## Abstract

This study advances knowledge concerning older immigrants’ cognitive health by examining the role of gender in the context of education, acculturation, and cognitive function. Guided by an emerging Gendered Process of Acculturation framework, we hypothesized that the cognitive health of men and women would benefit differently from a high level of education and acculturation. Cognitive health was measured with the Mini-Mental State Examination. Data were drawn from the Study of Older Korean Americans (SOKA), a multi-site survey of Korean Americans aged 60 and over (N = 2,061). Multivariate linear regression analyses were conducted to investigate the moderating role of gender in the effects of (1) education, (2) acculturation, and (3) the association of education and acculturation on cognitive function. Gender was found to be a significant moderator in the relationship of education and acculturation with cognitive health: the positive effect of both education and acculturation was greater among women than men. Furthermore, the three interaction among education, acculturation, and gender was significant: the positive impact of education on cognition was particularly pronounced among women with low acculturation and eliminated gender differences in cognitive status. Our findings suggest that gender plays a critical role in determining the cognitive health benefit arising from education and acculturation singularly and in concert, highlighting the vulnerability of women with low education and acculturation. These findings have implications for social interventions targeting immigrant populations.

